# Sharps injuries in a dental specialty hospital: retrospective analysis of occupational risks, 2020–2024

**DOI:** 10.1186/s12903-025-07020-z

**Published:** 2025-10-14

**Authors:** Mengqi Zhang, Yani Chen, Suna Zhang, Xiaolan Fan

**Affiliations:** 1 Infection Control Department, School and Hospital of Stomatology, Wenzhou Medical University, Wenzhou, 325000 Zhejiang P.R. China; 2 Endodontics, School and Hospital of Stomatology, Wenzhou Medical University, Wenzhou, 325000 China; 3 Pediatric Dentistry, School and Hospital of Stomatology, Wenzhou Medical University, Wenzhou, 325000 China

**Keywords:** Occupational exposure, Sharps injuries, Prevalence, Dentistry, Bloodborne

## Abstract

**Background:**

Occupational sharps injuries (OSI) remain a critical occupational health risk for healthcare workers, particularly in dental specialty settings where the use of fine instruments and frequent blood exposure heighten vulnerability. However, systematic data on high-risk populations, procedural factors, and pathogen distribution in dental specialty hospitals remain scarce. This study investigates the incidence, risk factors, and bloodborne infection profiles of sharps injuries in a tertiary dental hospital.

**Methods:**

A retrospective analysis was conducted on 170 OSI cases reported between January 2020 and December 2024 at a tertiary dental hospital in China. Data were extracted from paper-based registries and electronic adverse-event reporting systems, encompassing demographics, injury details, exposure sources, and pathogen profiles. Exclusion criteria included non-clinical injuries and incomplete records. Statistical analyses (SPSS 20.0) involved frequency calculations, chi-square tests, and Fisher’s exact tests (significance: *P* < 0.05).

**Results:**

A total of 170 OSI were reported between 2020 and 2024, with a annual incidence densities ranging from 3.09 to 5.15 per 100 person-years. Nurses (58.8%) and female workers (84.1%) constituted the majority of cases, while staff with ≤ 5 years of experience accounted for 64.7% of exposures. Bloodborne exposures dominated (76.5%), primarily caused by syringe needles (45.9%) and dental burs (9.4%). High-risk procedures included post-treatment instrument sorting (47.1%) and intraoperative handling (38.2%). Hepatitis B (9.2%) and syphilis (3.1%) were the most identified pathogens, though 63.85% of cases had unknown pathogen status. Dentists exhibited significantly higher hepatitis B exposure rates than nurses (13.9% vs. 5.4%, *P* = 0.04).

**Conclusion:**

This study highlights the urgent need for targeted interventions in dental settings, including enhanced training for nurses and early-career staff, optimized instrument-handling protocols, and mandatory pathogen screening for high-risk patients. These findings provide actionable insights to mitigate OSI risks and reduce bloodborne infection burdens in dental specialty hospitals.

**Supplementary Information:**

The online version contains supplementary material available at 10.1186/s12903-025-07020-z.

## Introduction

Occupational exposure refers to healthcare workers’ contact with hazardous substances or pathogens during diagnosis, treatment, or care delivery [1]. Such exposure typically involves contact with needles, sharp instruments, splashes, or contaminated materials through compromised skin or mucous membranes [2]. The United States Center for Disease Control and Prevention(CDC) defines sharps injuries as “a penetrating stab wound from a needle, scalpel, or other sharp object that may result in exposure to blood or other body fluids” [3]. Sharps injuries represent a major occupational health threat globally, particularly in dental specialty settings, where the use of delicate instruments (e.g., dental burs, suture needles) and confined operational spaces exacerbate risks [1–4]. A 2020 meta-analysis reported that 42.8% of nurses and 46.4% of physicians experienced sharps injuries [5], while single-institution studies found prevalence rates of 30%–58.8% among medical students [6–8]. Alarmingly, up to 80% of dentists and 61.9% of dental nurses report occupational exposure incidents, highlighting the urgency of addressing this issue in dental practice [9, 10].

Dental procedures involving high-frequency blood contact (e.g., tooth extraction, periodontal surgery) significantly increase exposure risks to bloodborne pathogens such as hepatitis B virus, syphilis, and Human Immunodeficiency Virus (HIV) [11, 12]. Despite proposed preventive strategies (e.g., safety-engineered devices, standardized protocols), their implementation in dental specialty hospitals remains suboptimal. For instance, a study in a Chinese tertiary dental hospital demonstrated that nonstandard operational behaviors contributed to repeated exposures [13]. Additionally, junior staff (≤ 5 years of experience) account for 71.2% of cases, underscoring the need for enhanced training and competency development [14]. Existing studies, however, predominantly rely on single data sources (e.g., electronic reporting systems), potentially underestimating exposure rates and overlooking multidimensional risk factors [13]. The unique environment of dental hospitals (such as complex types of instruments and high mobility of patients) requires more targeted risk assessment and intervention measures, but the related research is still in the exploratory stage.

By retrospectively analyzing data on occupational sharps injuries (OSI) in a tertiary care dental specialty hospital from 2020 to 2024, this study aims to extend the depth and breadth of existing research in the following key areas: first, to compare the risk of sharps injuries across different occupational roles; second, to identify temporal trends in exposure incidence; and third, to assess the types of bloodborne pathogens involved. These findings will inform evidence-based strategies to mitigate occupational risks and enhance infection control in dental specialty hospitals.

## Methods

This study follows the Declaration of Helsinki. Ethical approval was granted by the Medical Ethics Committee of School & Hospital of Stomatology Wenzhou Medical University (Approval No. WYKQ2025014). The study had no direct contact with the subjects and only collected information on occupational sharps injuries from hospital staff, which was used for analytical purposes only, so the Medical Ethics Committee of School & Hospital of Stomatology Wenzhou Medical University waived the informed consent of the subjects.

The study selected occupationally exposed persons with sharps injuries in the School & Hospital of Stomatology Wenzhou Medical University from January 1, 2020, to December 31, 2024, including dentist, nurse, medical technician, support staff, trainees. We summarized and compiled the occupational exposure registration forms completed through the paper version of the registration form in the hospital and the hospital’s medical safety (adverse) event reporting management system during the above time period, including basic information, description of the sharps injury exposure event, records of assessment of the exposure source’s history, test results, etc., and supplemented and verified the relevant information, and then summarized and analyzed the above information. This study covered all departments of the hospital. Our data collection was based on reports of sharps injury incidents at the hospital, and all sharps injury incidents were reported voluntarily by the relevant personnel. The study excluded injuries that occurred outside the hospital or were not related to clinical work, as well as data with unclear or incomplete records.

The study was conducted at the School & Hospital of Stomatology, Wenzhou Medical University—a tertiary dental hospital providing comprehensive dental services, including: Oral and Maxillofacial Surgery, Pediatric Dentistry, Endodontics, Prosthodontics, Orthodontics, Periodontics, General Dentistry. The term ‘dentists’ is used throughout to refer to all clinical dental specialists (including oral surgeons, pediatric dentists, endodontists, etc.). Annual incidence density was calculated as: (Number of OSI/Total person-years at risk)×100, where total person-years equaled the mean number of active staff multiplied by observation time (1 year). Quarterly incidence density used person-quarters as the denominator. The categories of personnel in this study were categorized as follows: dentists, nurses, medical technicians, support staff (including caregivers, housekeepers, and sterilizers), and trainees. Dental nurses are licensed professionals responsible for: clinical chairside assistance; instrument sterilization and tray preparation; biohazard waste management; and infection control compliance. Their role involves extensive handling of contaminated sharps during post-procedural cleanup. We categorized the types of OSI into bloodborne occupational exposures and non-bloodborne occupational exposures. Bloodborne Occupational Exposure (BOE) refers to sharps injuries that come into contact with blood, body fluids, or contaminated instruments that may contain pathogens. Non-Bloodborne Occupational Exposure (NBOE) refers to sharps injuries that do not come into contact with blood or infectious body fluids, but only involve clean instruments or non-infectious substances. We classified the timing of OSI into five categories: preparing supplies before treatment, during treatment, sorting out supplies after treatment, recycling of medical waste, and cleaning and sterilizing. Preparing supplies before treatment refers to the exposure that occurs when a healthcare worker is preparing supplies such as examination trays and technician’s forceps boxes prior to treatment or care of a patient. During treatment refers to the exposure that occurs when a healthcare worker is performing dental treatment or care for a patient. Sorting out supplies after treatment refers to the exposure that occurs when healthcare workers organize and dispose of supplies after treating and caring for patients. Recycling of medical waste refers to the exposure that occurs when cleaning staff recycle and dispose of medical waste. Cleaning and sterilizing refers to exposures that occur when pre-treating instruments or sterilizing surfaces.

Data were analyzed using SPSS 20.0 (IBM, USA). Categorical variables were summarized as frequencies and percentages. Between-group differences (e.g., occupation-specific injury rates, pathogen distributions) were assessed using chi-square tests or Fisher’s exact tests. Fisher’s exact test was employed when expected frequencies in contingency tables were < 5; otherwise, Pearson’s chi-square test was used. Statistical significance was defined as *P* < 0.05.

## Results

**Fig. 1:**
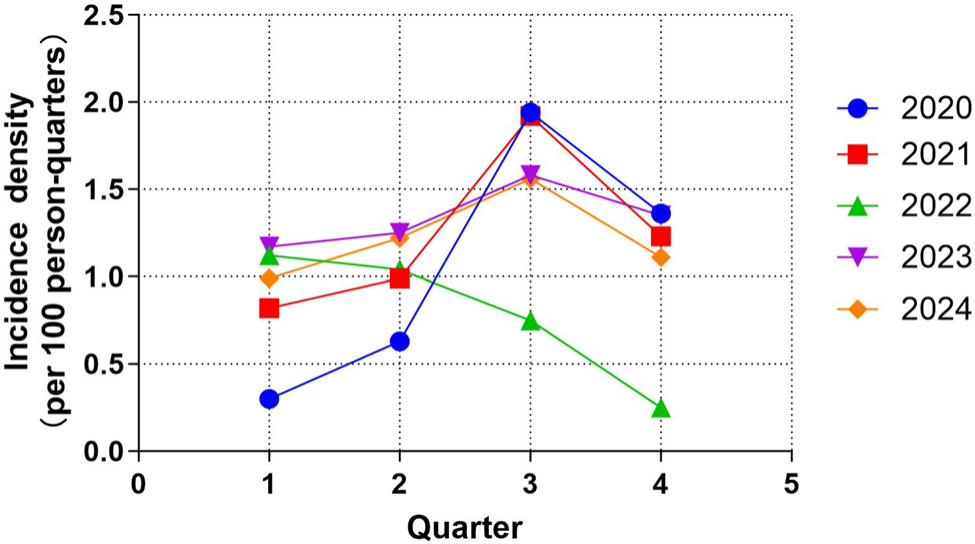
Quarterly incidence density (per 100 person-quarters) of occupational sharps injuries

A total of 170 OSI occurred from January 1, 2020 to December 31, 2024, with annual incidence densities ranging from 3.1 to 5.2 per 100 person-years, as shown in Table 1. The annual distribution of occupational categories among hospital staff shows that nurses account for the highest proportion (28.9%–30.1%), followed by dentists (27.2%–28.9%) (Table S1).Statistical analyses of 170 OSI were conducted quarterly for each year, and except for a decreasing trend in the number of occurrences in 2022, the results of the rest of the years showed year-round fluctuations, with the incidence peaks were all concentrated in the third quarter (July to September) (Fig. 1).

**Table 1:** Annual incidence density (per 100 person-years) of occupational sharps injuries

Year	Variable			
	Number of consultations	Number of occurrences	Total personnel	Annual incidence density (per 100 person-years)
2020	358,801	28	662	4.8
2021	472,736	36	734	5.2
2022	463,057	25	810	3.1
2023	501,124	42	815	4.9
2024	531,992	39	813	4.2

Nurses (58.8%) and female workers (84.1%) constituted the majority of OSI, with staff having ≤ 5 years of experience accounting for 64.7% of exposures. Most incidents involved bloodborne occupational exposures (76.5%). Syringe needles were the predominant exposure source (45.9%), followed by dental burs (9.4%) (Table 2).

**Fig. 2:**
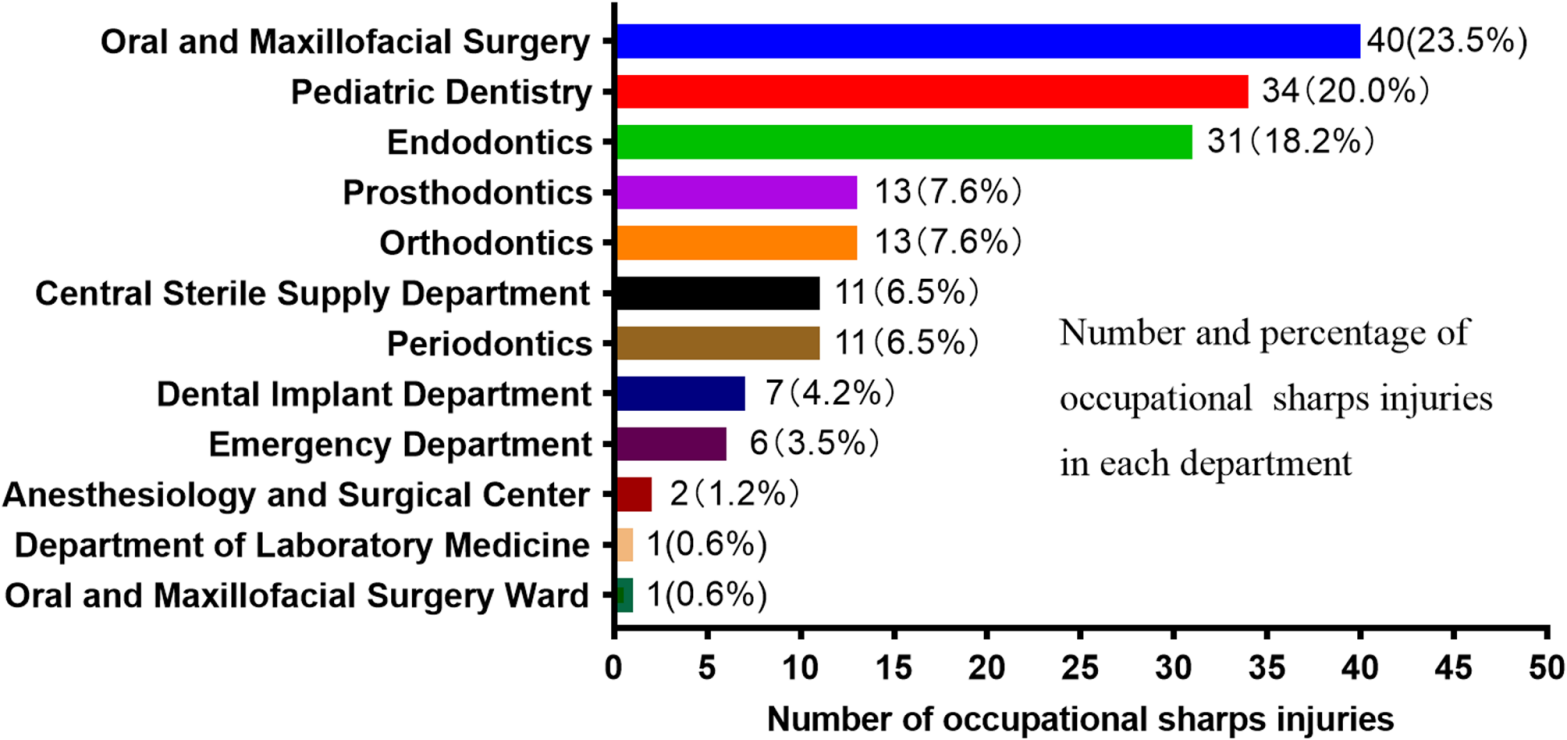
Distribution of departments with occupational sharps injuries in the dental hospital

**Fig. 3:**
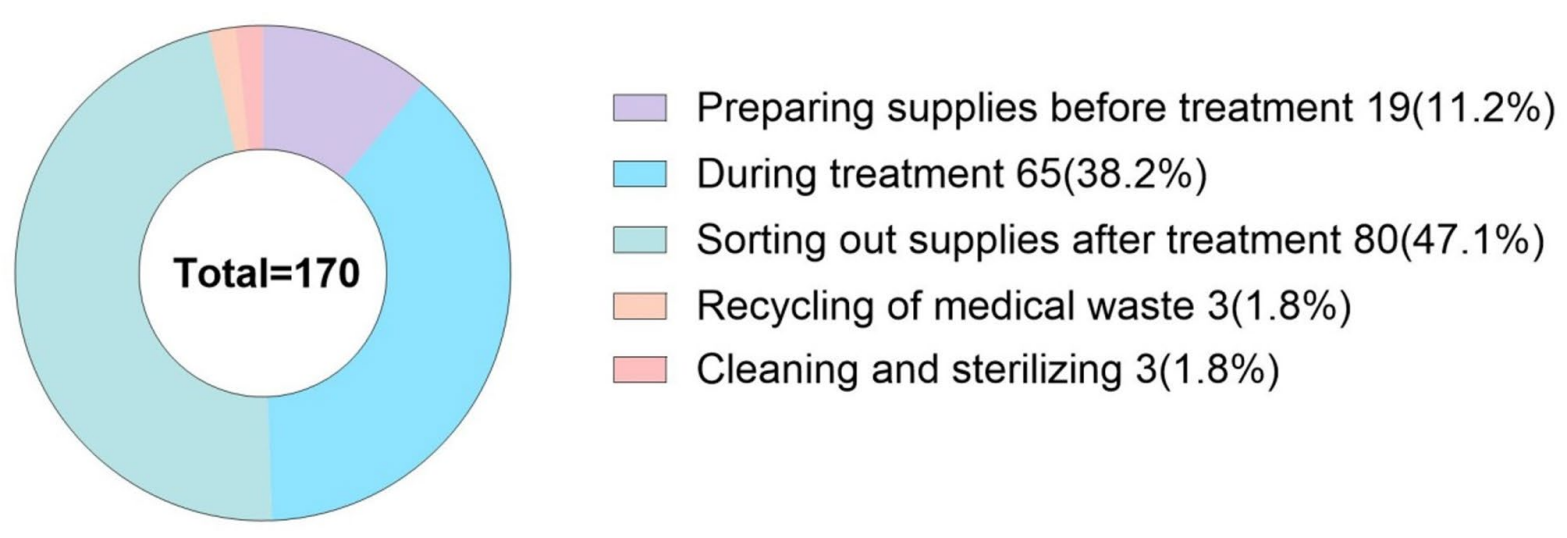
The proportion of different operation links when occupational sharps injuries occurred

**Fig. 4:**
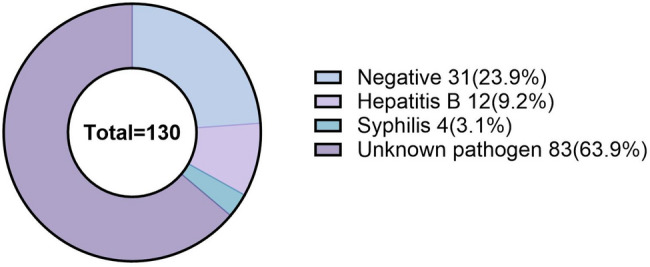
The proportion of different pathogens in occupational exposure to blood-borne sharp instrument injury

The departments with the highest incidence of OSI were Oral and Maxillofacial Surgery (23.5%), Pediatric Dentistry (20.0%), and Endodontics (18.2%) (Fig. 2). The majority of injuries occurred when sorting out supplies after treatment (47.1%) and during treatment (38.2%) (Fig. 3). Data from 130 bloodborne OSI showed that the major bloodborne infection in occupational exposures of hospital workers was Hepatitis B (9.2%), followed by Syphilis (3.1%), with an additional 63.9% of cases originating from patients with uncertain pathogen status (Fig. 4).

**Table 2:** The basic situation of occupational sharps injuries among hospital staff from 2020 to 2024

Variable	*N* (%)
Sex	
Man	27/170 (15.9)
Woman	143/170 (84.1)
**Occupational category**	
Dentist	45/170 (26.5)
Nurse	100/170 (58.8)
Medical technician	1/170 (0.6)
Support staff	7/170 (4.1)
Trainees	17/170 (10.0)
**Length of service**	
≤ 1 year	53/170 (31.2)
1 ~ ≤ 5 years	57/170 (33.5)
5 ~ ≤ 10 years	29/170 (17.1)
> 10 years	31/170 (18.2)
**Types of occupational exposure**	
Blood-borne occupational exposures	130/170 (76.5)
Non-blood-borne occupational exposures	40/170 (23.5)
**Types of exposure source**	
Syringe needle	78/170 (45.9)
Dental bur	16/170 (9.4)
Suture needle	15/170 (8.8)
Dental probe	10/170 (5.9)
Endodontic file	9/170 (5.3)
Glass ampoule	6/170 (3.5)
Excavator	4/170 (2.4)
Orthodontic archwire	3/170 (1.8)
Porcelain-fused-to-metal crown	3/170 (1.8)
Surgical scalpel	3/170 (1.8)
Dental elevator	3/170 (1.8)
Other sharps	17/170 (10.0)

Fisher’s exact probability test (Table 3) showed that there was a statistically significant difference in the distribution of pathogens between occupational categories (*P* = 0.04). Among all pathogens, exposure to hepatitis B was predominant among healthcare workers, followed by syphilis. It is noteworthy that the percentage of unknown pathogens was higher in all occupational categories. Fisher’s exact probability test was also used to analyse the association between years of work experience and types of pathogens. The results showed that there was no statistically significant difference in the distribution of pathogens between the different years of experience groups (*P* = 0.41) (Table 3).

**Table 3:** Pathogen status of exposure sources in bloodborne Sharp injuries among staff with different work experience and occupational categories (*N* = 130)

Variable	Exposure pathogens						*P* value
	Negative	Hepatitis B	Hepatitis C	HIV/AIDS	Syphilis	Unknown pathogen	
**Occupational category**							
Dentist	14/36 (38.9)	5/36 (13.9)	0/36 (0.0)	0/36 (0.0)	1/36 (2.8)	16/36 (44.4)	0.04*
Nurse	13/74 (17.6)	4/74 (5.4)	0/74 (0.0)	0/74 (0.0)	2/74 (2.7)	55/74 (74.3)	
Medical technician	0	0	0	0	0	0	
Support staff	0/6 (0.0)	0/6 (0.0)	0/6 (0.0)	0/6 (0.0)	0/6 (0.0)	6/6 (100.0)	
Trainees	4/14 (28.6)	3/14 (21.4)	0/14 (0.0)	0/14 (0.0)	1/14 (7.1)	6/14 (42.9)	
**Length of service**							
≤ 1 year	8/47 (17.0)	4/47 (8.5)	0/47 (0.0)	0/47 (0.0)	1/47 (2.1)	34/47 (72.3)	0.41
1 ~ ≤ 5 years	8/36 (22.2)	4/36 (11.1)	0/36 (0.0)	0/36 (0.0)	3/36 (8.3)	21/36 (58.3)	
5 ~ ≤ 10 years	9/25 (36.0)	2/25 (8.0)	0/25 (0.0)	0/25 (0.0)	0/25 (0.0)	14/25 (56.0)	
> 10 years	6/22 (27.3)	2/22 (9.1)	0/22 (0.0)	0/22 (0.0)	0/22 (0.0)	14/22 (63.6)	

Data reflect pathogens identified in the source patients of occupational exposures, not seroconversion outcomes in healthcare workers

Abbreviations: *P*, probability

*Significance: *P* < 0.05

Incidence of OSI among dentists by year showed a decline in 2020–2022, recovered in 2023 and peaked in 2024. Injury rates for nurses fluctuated, peaking at 11.3 per 100 person-years in 2021 and declining in 2024 (Table 4). As shown in Table 4, the injury rate for nurses was 11.3 per 100 person-years in 2021, which was significantly higher than that for dentists at 3.0 per 100 person-years (Pearson χ2 = 9.9, *P*<0.01). The difference between the injury rate for nurses (6.0 per 100 person-years) and that for dentists (2.3 per 100 person-years) in 2022 was still statistically significant (Fisher exact test, *P* = 0.04). Of the other years, the injury rate of nurses was still higher than that of dentists in 2020 (*P* = 0.05) and 2023 (*P* = 0.08), although the significance threshold was not reached, and there was no significant difference between the two groups in 2024 (Pearson χ2 = 0.7, *P* = 0.41).

**Table 4:** Annual occupational sharps injuries among dentists and nurses

Year	Dentist			Nurse			Pearson χ²	*P* value
	Total number of dentists	Number of injured dentists	Injury rate	Total number of nurses	Number of injured nurses	Injury rate		
2020^a^	191	8	4.2	199	18	9.1		0.05
2021^b^	203	6	3.0	221	25	11.3	9.9	<0.01*
2022^a^	221	5	2.3	234	14	6.0		0.04*
2023^a^	222	12	5.4	247	24	9.7		0.08
2024^b^	229	14	6.1	245	19	7.8	0.7	0.41

Abbreviations: *χ²*, chi-square; *P*, probability; The unit of injury rate is per 100 person-years. Fisher’s exact test was used when expected frequencies were < 5; otherwise, Pearson χ² was applied

*Significance: *P* < 0.05

^a^ Fisher’s exact test was used ^b^ Pearson χ² was applied

## Discussion

By retrospectively analyzing the data on OSI in a tertiary dental specialty hospital from 2020 to 2024, this study reveals the high-risk groups, key aspects, and pathogen distribution characteristics of occupational exposures in the field of dentistry, which provides an important basis for optimizing protection strategies.

The incidence of OSI rebounded in 2023 after a brief decline in 2022, possibly related to fluctuations in consultation volume after the COVID-19 outbreak. The peak exposure in the third quarter (Fig. 1) may be associated with the surge in patient volume and increased work intensity of healthcare workers during the summer months, suggesting the need for additional manpower and enhanced protective supervision during peak periods.

The group of nurses (58.8%) and employees with ≤ 5 years of service (64.7%) were the main exposed population for OSI, a finding consistent with several studies [14–16]. A nine-year study on work-related injuries in Turkey revealed that nurses had the highest rate of workplace injuries, with 78.2 cases per 1,000 nurses, followed by 26.8 cases per 1,000 administrative and laboratory technicians, and 16.9 cases per 1,000 doctors [17]. A survey in the United States indicated that nurses reported the highest proportion of sharps injuries (71%), while medical students reported the lowest proportion (40%) [3]. The high injury rate among nurses may be related to their frequent operations in instrument sorting and medical waste recycling, which multiply the risk due to multiple exposures involving sharp instruments. However, there are different findings, such as the results of a ten-year retrospective study in a tertiary care hospital in Southeast Asia showed that physicians were the group that reported the most OSI [18]. A Japanese study conducted a statistical analysis of 19 years of data from a hospital, showing that among all needle stick and sharps injuries (NSIs) that occurred, dentists accounted for 45.1%, residents and students 26.7%, central supply technicians 12.3%, nurses 9.7%, dental hygienists 4.6%, and others 1.5% [19]. This may be due to differences in geographic location and hospital type, making the results different between individual general hospitals and specialized dental hospitals.

In addition, the injury rate of low-working-age employees was significantly higher than that of the high-working-age group, suggesting that new recruits may have an increased number of errors due to inexperience in operation, insufficient training, or psychological tension [20, 21]. This is consistent with the results of previous studies, which found that low working-age employees have significant shortcomings in the standardization of operating procedures and awareness of protection [15, 16]. In the future, simulation training for this group needs to be strengthened and combined with psychological counseling to reduce the impact of anxiety on operational safety. In terms of exposure sources, syringe needles were the most common injury-causing instrument (45.9%), followed by Dental bur (9.4%), which is consistent with the results of previous studies [10, 22]. Since these oral instruments are very slender and can easily puncture the personal protective equipment, they are not effective in avoiding OSI even after proper wearing of personal protective equipment. When operating in an oral specialty, it will be frequently encountered that when anesthesia is applied during treatment, the nurse sometimes helps the dentist to recap the needle, and sometimes it is necessary to break and bend the needle, which undoubtedly increases the risk of exposure [10, 23]. The predominance of female injuries (84.1%) reflects the gendered distribution of nursing roles in Chinese healthcare, where 89–95% of nurses are female [14, 15, 24]. This pattern—coupled with nurses’ high injury risk—explains the observed sex disparity despite the lack of institutional sex-composition data.

The highest incidence occurring sorting out supplies after treatment (47.1%) and during treatment (38.2%) (Fig. 3). This phenomenon is in line with previous studies, pointing out the complexity of the instrument handling process after dental consultation (e.g., separating instruments with both hands, poor placement of sharps boxes) as a key cause of exposure [25]. At the same time, a large body of evidence suggests that manipulations such as two-handed needle recapping, bending, and removing needles are the most common causes of OSI in oral practice [21, 26, 27]. In addition, protective equipment factors (e.g., insufficiently equipped sharps boxes, inconvenient access to personal protective equipment) accounted for the next highest number of cases, suggesting that hospitals need to optimize the layout of equipment and regularly check the availability of protective equipment. For the high incidence areas, it is recommended to introduce one-handed operation techniques or automated instrument retrieval systems to reduce human errors.

Hepatitis B (9.2%) and syphilis (3.1%) were the major pathogens in bloodborne exposures, but the pathogen status was unknown in 63.9% of the cases (Fig. 4), which may be related to the low rate of testing of patients at the source of the exposures or the lack of records. Since most of the dental consultations are conducted in outpatient clinics, patients are mostly not tested for bloodborne diseases, thus staff members perform consultation and care operations under unknown conditions, which greatly increases the risk of bloodborne exposure. In recent years, the incidence of blood-borne diseases has increased, and studies have shown that the detection rates of hepatitis C virus and AIDS in trauma patients have reached 14.0% and 13.5%, respectively [28]. Previous studies have shown that the pre-treatment screening rates for hepatitis B virus, hepatitis C virus, HIV and syphilis in dental cleaning patients were 5.2%, 1.1%, 0.2% and 0.4%, respectively [29]. The high percentage of unknown pathogens highlights the importance of improving the tracking mechanism of exposure sources. To reduce the risk posed by unknown pathogen status (accounting for 63.9% of exposure cases), we recommend that HIV, HBV, and HCV testing be performed immediately on the source patient for any sharps injury incident. Test results should be recorded in the patient’s medical record to guide the rapid implementation of post-exposure prophylaxis.

The significant difference in pathogen distribution between occupations (*P* = 0.04) reflect differences in the ability to trace sources of exposure. Dentists sustained 72.2% (26/36) of injuries during patient treatment where sources were identifiable, enabling HBV testing in 55.6% (20/36) of cases. Conversely, nurses incurred 68.9% (51/74) of injuries during instrument sorting/waste handling where sources were untraceable, resulting in 74.3% (55/74) unknown pathogen status (Table 3). The significantly higher HBV rate among dentists (13.9% vs. 5.4% in nurses) is an artifact of differential source tracing.

Oral and maxillofacial surgery (23.5%), pediatric dentistry (20.0%), and endodontics (18.2%) were the departments with the highest incidence of sharp instrument injuries (Fig. 2). Oral and maxillofacial surgery requires frequent use of small, sharp instruments such as files, dental drills, probes, syringe needles, razor blades, and suture needles due to the involvement of complex surgical operations (e.g., bone cutting, suturing), and the characteristics of frequent instrument use and restricted operating space increase the risk of exposure [4, 12]. A study in South Africa showed that the department with the highest incidence of needlestick injuries was oral and maxillofacial surgery and that the majority of OSI were caused by needlestick injuries, which is consistent with our findings [24]. The high exposure rate in pediatric dentistry may be related to uncontrolled instrumentation due to low child cooperation, and it is recommended that behavioral management techniques (e.g., sedation therapy or parental involvement in calming) be introduced to minimize operative interference. In addition, endodontics requires a high degree of precision due to the use of fine instruments (e.g., root canal files), and intensive training in microscopic manipulation and the promotion of safety instruments are needed.

In the annual analysis, nurses had higher incidence of OSI than dentists in 2020–2023 (Table 4).This disparity may be attributed to nurses’ high-frequency involvement in instrument reprocessing and medical waste handling—procedures involving uncontrolled sharps with elevated injury risk. Dentists primarily experience exposure during controlled clinical procedures (e.g., injections, extractions), whereas nurses face cumulative risks during post-treatment sorting, needle recapping, and waste disposal where situational hazards (e.g., rushed workflows, obscured sharps) compound injury likelihood. Notably, the statistically non-significant difference in 2024 (7.8% vs. 6.1%, *P* = 0.41) may reflect recent interventions: enhanced safety training for nursing staff on sharps handling protocols, standardization of double-gloving during waste processing, and hospital-wide adoption of safety-engineered devices (e.g., retractable syringe needles) since 2023. In addition, the difference in Incidence of OSI between nurses and dentists reflects the operational characteristics of the different occupations, and differentiated protection strategies need to be developed, such as focusing on optimizing the instrument handling process for nurses and intensive blood exposure protection training for dentists.

Limitations remain in this study. First, the data relied on a passive reporting system, which may have omissions or information bias. Second, the effects of psychological factors on operational safety were not included. Third, the absence of sex-stratified workforce data precluded calculation of sex-specific injury rates. Future studies should collect full demographic denominators to enable refined risk analyses. Repeated injuries are also a research topic worth considering in the future. In addition, multicenter studies are recommended to validate the generalizability of the present findings and to assess the actual effects of the interventions.

## Conclusions

This analysis provides practical insights for sharps injury prevention in dental specialty hospitals. Nurses and early-career staff face significantly higher injury risks than other occupational groups, indicating that targeted interventions should prioritize these high-risk personnel. Additionally, while seasonal workload surges and procedural settings present uniformly elevated hazards, systemic gaps in exposure source tracking and staff immunization demand institutional solutions. Sustained vigilance toward these risks remains essential for effective infection control.

## Supplementary Information

Supplementary Material 1.

## Data Availability

Data will be made available on reasonable request.

## Abbreviations

OSI: Occupational sharps injuries
CDC: Center for disease control and prevention
HIV: Human immunodeficiency virus
BOE: Bloodborne occupational exposure
NBOE: Non-bloodborne occupational exposure
