# Supplementary material for: Sharps injuries in a dental specialty hospital: retrospective analysis of occupational risks, 2020–2024
Source: BMC Oral Health. 2025 Oct 14;25:1618. doi: 10.1186/s12903-025-07020-z (PMC12523194; doi:10.1186/s12903-025-07020-z)
Supplement: Supplementary file 1 — Supplementary Material 1. [file 12903_2025_7020_MOESM1_ESM.docx]

| **Table S1.** The occupational composition of hospital staff (2020–2024) | | | | | | | |
| --- | --- | --- | --- | --- | --- | --- | --- |
| Year | Total | Dentist,  n (%) | Nurse,  n (%) | Medical technician,  n (%) | Support staff, n (%) | Trainee, n (%) |  |
| 2020 | 662 | 191 (28.9) | 199 (30.1) | 46 (6.9) | 100 (15.1) | 126(19.0) |  |
| 2021 | 734 | 203(27.7) | 221(30.1) | 48(6.5) | 122(16.6) | 140(19.1) |  |
| 2022 | 810 | 221(27.3) | 234(28.9) | 51(6.3) | 154(19.0) | 150(18.5) |  |
| 2023 | 815 | 222(27.2) | 247(30.3) | 52(6.4) | 152(18.7) | 142(17.4) |  |
| 2024 | 813 | 229 (28.2) | 245 (30.1) | 52 (6.4) | 139 (17.1) | 148(18.2) |  |
